# Gender Typicality and Engineering Attachment: Examining the Viewpoints of Women College Engineers and Variation by Race/Ethnicity

**DOI:** 10.3390/bs14070573

**Published:** 2024-07-06

**Authors:** Ursula Nguyen, Catherine Riegle-Crumb

**Affiliations:** 1 Department of Teaching, Learning, and Teacher Education, University of Nebraska—Lincoln, 840 N 14th St., Lincoln, NE 68588, USA; 2 Department of Curriculum and Instruction, STEM Education, The University of Texas at Austin, 1912 Speedway, Stop D500, Austin, TX 78712, USA; riegle@austin.utexas.edu

**Keywords:** gender typicality, engineering, race/ethnicity, higher education

## Abstract

Women remain under-represented in many STEM occupations, including in the high-status and lucrative field of engineering. This study focuses on women who have chosen to enter this men-dominated field, to consider whether and how feelings of gender typicality predict their attachment to the field. Specifically, utilizing a U.S. sample of approximately 800 women college engineers from diverse racial/ethnic backgrounds, we build on emerging research on gender typicality to distinguish perceptions of feminine typicality as well as masculine typicality. Subsequently, we consider whether these perceptions have implications for their attachment to engineering, including their engineering identity as well as their certainty of staying in the field. Importantly, in doing so, we consider potential racial/ethnic variations in these relationships.

## 1. Introduction

Despite decades of efforts towards increasing women’s representation in engineering, this high-status STEM field remains men-dominated. Less than 25% of U.S. undergraduate degrees are attained by women, and an even smaller percentage of women work in the engineering labor force in the U.S. [1]; additionally, the under-representation of Black/African-American, Latinx/Hispanic, and Indigenous women is even more pronounced, as they comprise only about 5% of bachelor’s degree holders in engineering [1]. While the relatively low likelihood that young women choose engineering majors in college largely contributes to this under-representation (and consequently, a large body of extant research investigates the reasons behind this (e.g., see Cheryan and Markus [2] and Xie et al. [3])), attrition remains a significant concern even among women engineering majors, as many choose to switch majors or not transition to the engineering labor force after college. Therefore, in this study, we consider factors which may facilitate college women’s persistence or attachment to engineering.

Specifically, utilizing a sample of over 800 college women engineers, we consider how they define their gender typicality, and whether and how this may have implications for their engineering attachment. At a cursory first glance, one might expect that women in engineering might perceive themselves as gender atypical, as they have chosen a field that is dominated by men. But such a view does not recognize that gender itself is a multi-dimensional social construct [4], and as such, for example, individuals can simultaneously feel identification with women and with men. And while research on gender inequality too often implicitly assumes that women in STEM fields constitute a relatively homogenous group, we draw on a newly emerging body of social-psychological research [5], to consider variations in how women engineers perceive themselves in relation to other women (feminine typicality) and how they perceive themselves in relation to men (masculine typicality), and the potential implications of both for their attachment to engineering.

Given the hegemonically masculine culture associated with the field of engineering, it seems likely that self-perceptions of masculine typicality may be related to higher attachment to engineering for women engineers. Yet it is not necessarily clear whether and how feminine typicality might relate to engineering attachment when it is viewed as its own distinct identity, and not simply the polar opposite of masculinity. Indeed, we note that most related research has either focused on affinity or typicality to one gender (e.g., femininity) or considered gender typicality or gendered traits on a continuum from masculine to feminine (e.g., references [6,7]). Therefore, our study will make a contribution to research on women’s under-representation in STEM by moving beyond a typically simplistic definition of gender as one singular identity.

Additionally, as we recognize that gender identity is nuanced and complex, we also recognize that gender intersects with racial/ethnic identities [8,9]. Specifically, while women are marginalized within engineering, experiences are not uniform across race; for example, Black and Latinx women often endure a solitary experience of being one of few or the only one in their field, while White women benefit from racial privilege [10,11,12]. Further, definitions of ‘typical’ gender behaviors and the performance of masculinity and femininity vary across communities, with potential implications not only for how women of different racial/ethnic identities view their own gendered selves but also for how they see themselves as ‘fitting’ in engineering spaces. 

As such, two research questions guide our study. First, how do women college engineers define their gender typicality along feminine and masculine dimensions, and how does this vary by race/ethnicity? Second, how do their perceptions of feminine typicality and masculine typicality predict their attachment to engineering, including their engineering identity and intentions to stay in the field, and how does this vary by race/ethnicity? In investigating these research questions, this study seeks to advance our knowledge of the complexity of ways that gender identities may shape the experiences of those under-represented within men-dominated fields.

## 2. Theoretical Background

### 2.1. Gender as a Multi-Dimensional Social Construct

From a sociological lens, gender scholars recognize gender as a social construction that operates across levels, including the institutional level, the interactional level, and the individual level [4,13]. Gendered beliefs and stereotypes reverberate throughout levels of the system, creating inequality via shaping roles, norms, and expectations. This system is fundamentally hierarchical, as what is defined as masculine (which can shift over time and place) is socially valued as having higher status. Within the contemporary gender system of the U.S., STEM fields, such as engineering, are culturally defined as masculine, and as such, stereotypes, norms, and expectations function to preserve the space as dominated by men [2,14].

Yet while prevailing cultural narratives still pose men and women as distinct gender groups, individuals can and do define themselves in ways that do not always neatly adhere to such categorical distinctions. Indeed, many individuals, such as those who identify as gender fluid or transgender, reject the gender binary and/or related assumptions that biological sex and gender are synonymous [15]. The recognition that individuals’ lived experiences of gender identity are highly complex and multi-dimensional has informed considerable social psychological research. 

For example, recent theoretical work by Martin and colleagues [5] has expanded the notion of gender typicality, originally posited by Egan and Perry [16], as one crucial dimension of gender identity that captured the sense of compatibility or ‘fit’ with one’s gender (i.e., own-gender typicality), to further acknowledge that individuals likely feel varying levels of similarity to more than one gender. This perspective of gender identity as multi-dimensional allows us to explore how individuals view themselves as performing roles and behaviors that are culturally associated with femininity and masculinity and to consider the ways in which each may be recognized or valued differently depending on the context. As we will discuss further in subsequent sections, regarding the specific case of women in engineering, examining their gender identity in terms of both feminine and masculine typicality is important, as it may have implications for their participation and inclusion in a hegemonically masculine space.

### 2.2. The Intersection of Gender and Race

Our study is also informed by insights from intersectional scholars, who call attention to the co-construction and intersection of systems of race and gender [17,18]. An intersectional lens emphasizes the relational and dynamic aspects between social positions that are rooted in power structures of inequality, such as racism and sexism, with White men occupying the position with the most power and prestige [18,19,20]. Further, the construction of gender is interdependent with race, such that gendered norms, stereotypes, and beliefs can vary across different racial/ethnic communities. 

While theories and related empirical work on gender typicality have only recently begun to explore and unpack this reality, we note that prior research on gender roles has suggested that notions of masculinity, as defined by the privileged White community of European ancestry, may not necessarily be seen as at odds with femininity within Black communities [21,22]. Further, cultural definitions of appropriate gender roles for women in both Latinx and Asian communities may be tightly circumscribed and comparatively restrictive compared to other communities [23,24]. As we discuss further below, among our sample of women college engineers, we will explore how feminine typicality and masculine typicality may diverge across racial/ethnic groups, and also may have different implications for their attachment to their chosen field.

## 3. Literature Review

As mentioned previously, research on gender typicality as an important dimension of gender identity is comparatively recent and has primarily focused on adolescent well-being as an outcome of interest [5,25], with only a few studies considering its potential links to gender inequality in STEM spaces [26,27]. In the sections below, we briefly review this literature. As gender typicality has not yet been examined among young women engineers, we then turn to discuss prior research that has—in some way—examined how aspects of femininity or of masculinity may impact women’s experience in engineering. Doing so helps inform what we might find when directly measuring how self-perceptions of feminine typicality and masculine typicality predict women engineers’ attachment to their field. Finally, throughout the sections below, we note the few instances of research that explore racial/ethnic variation, as our intention for this study is to provide a much-needed step forward in research in this area.

### 3.1. Previous Empirical Work on Own-Gender Typicality

Seminal work by Egan and Perry [16], emphasized gender typicality as an important dimension of gender identity. They created what has since become a widely used scale to measure how similar people felt to others of their own gender; items ask respondents to rate their level of agreement with various statements about feeling “just like” people of their gender [16] (p. 463). Research in this area generally finds that feeling similar to those of your gender group is associated with higher self-esteem, particularly for older children and adolescents [16]. Studies utilizing the scale developed by Egan and Perry [16] have expanded into the higher education space as well. For example, work by Wilson and Leaper [25] and DiDonato and Berenbaum [28] utilized samples of college students, and found that among men and women, feeling more gender-typical was associated with significantly higher self-esteem. In general, this body of research on own-gender typicality finds that how individuals feel that they ‘fit’ with their (chosen or assigned) gender positively predicts their psychological well-being. Among these studies, only Wilson and Leaper [25] explore racial/ethnic variation and find that the association between own-gender typicality and self-esteem is stronger for Asian women than for either White or Latinx women.

A handful of studies have considered how own-gender typicality might have implications for young people’s attitudes and choices regarding domains that are highly populated or associated with one gender, such as STEM fields. Specifically, there is some evidence that for young women, higher levels of own-gender typicality could deter them from developing interest and confidence in men-dominated STEM fields. For example, using a sample of U.S. middle school students, John and colleagues [26] found that among girls, higher own-gender typicality was associated with higher math anxiety, while among boys, higher own-gender typicality was associated with lower math anxiety. Relatedly, another study of young women college students in Thailand found that those who reported higher levels of own-gender typicality had correspondingly lower self-efficacy in computer science [29]. Finally, in another study of college students at one university in the U.S., Dinella et al. [6] found that women with higher own-gender typicality expressed less interest in men-dominated careers like STEM. We note that none of these studies considered the potential for racial/ethnic variation.

### 3.2. Research Examining Feminine Typicality and Masculine Typicality

While the research cited above finds evidence that own-gender typicality is important to consider (and predicts generally positive social-psychological outcomes, but perhaps less interest in men-dominated fields among young women), it does not consider the potential for simultaneous identification with a different gender group. To rectify this narrow view of gender identity, Martin and colleagues [5] proposed that researchers should examine both feminine typicality and masculine typicality—what they referred to as dual gender typicality. In their sample of children, they correspondingly developed scales to measure each, and confirmed that they represented distinct and unique dimensions of gender identity that were only moderately negatively correlated with one another, such that perceived masculinity and femininity should not be considered “mirror images of one another” [5] (p. 172). Further, their results indicated that among both boys and girls, *both* masculine and feminine typicality were associated with positive social-psychological outcomes (e.g., less socially anxious, more connections with peers). A recent study of college students used the same measures and came to similar conclusions, namely that among both young men and young women, better psychological adjustment was promoted by higher levels of *both* masculine and feminine typicality [30]. Interestingly, a study by Nielson et al. [31] of a sample of sixth-grade students provides evidence of the potentially negative implications when young people only feel typical of their own gender (but not typical of another gender), as this was associated with high levels of felt pressure to conform to gender norms. The authors also found a substantial portion of their sample reported being highly typical of both boys and girls; however, this pattern was much more common among girls. Such work prompts reflection about whether contemporary cohorts of young people may be somewhat more flexible in their view of gender roles than perhaps in previous generations. However, a clear limitation of the results within this emerging research area is the lack of consideration of possible divergence by race/ethnicity—a limitation we seek to address.

### 3.3. Considering Women in STEM 

In this study, we build on the work described above [5,30] to explore variations in gender identity among women college engineers, in the form of identification with feminine typicality and masculine typicality, and consider potential implications for their attachment to the field. In doing so, we are also informed by a wealth of prior research on women in men-dominated STEM fields, which has explored how women feel a lack of fit in such fields, or examined how various traits or characteristics that are typically associated with one gender may be valued (or devalued) in these spaces.

For example, prior quantitative research in this area often measures what is referred to as ‘woman-scientist identity interference’ or the perceived incompatibility between one’s gender and one’s scientific identity for women in STEM majors. Research finds that such interference is related to a lower sense of well-being and lower perceived performance [32,33]. At the same time, those who viewed being a woman as compatible with a scientific identity had higher levels of belonging and reported a lower likelihood of dropping out of their major [34,35,36]. Further, some of these studies find that the positive relationship between identity compatibility and women’s sense of belonging does not differ by race/ethnicity [35,36]. While certainly informative, this research is nevertheless limited by treating women’s gender identity as singular, as women are asked to report their level of agreement with items such as “I feel that other scientists do not take me seriously because I am a woman” [32] (p. 491). In other words, gender identity is treated as a category—you are a woman or you are not. Therefore, likely variations in women’s feelings of typicality with their gender are lost, and among a group of women who have chosen to enter men-dominated spaces, also considering their identification with men is clearly warranted.

Indeed, qualitative literature on women in STEM has considered the ways in which women may perform masculinity as a coping mechanism or way to fit in within hegemonically masculine spaces. For example, a study of women engineering majors by Powell et al. [37] found that “women are assimilated into the masculine engineering cultures through processes of gender performance” (p. 425), which included “acting like one of the boys” (p. 418) as well as disavowing their femininity. Further, a few studies find that some young women who self-identify as being more tomboyish view STEM as a possible career for them and develop a strong STEM identity [38,39,40]. Further, while the qualitative literature certainly points to some advantages for women in men-dominated STEM spaces who enact masculinity, it also suggests that positive identification as a STEM person may be possible for young women who can flexibly navigate between femininity and masculinity [38,41]. For example, one study found that women engineers viewed asking for help and working together as knowingly feminine traits but also saw them as having value in getting ahead [37]. 

Thus, the deeper exploration of gender identity found in some qualitative work is consistent with the focus of this study, which recognizes the importance of a multi-dimensional conceptualization of gender identity, such that women can feel and enact both feminine typicality and masculine typicality. As such, we explore the implications of both for women’s attachment to engineering, including their engineering identity and their intentions to continue in the field. Further, a focus on potential variations in these relationships by women’s race/ethnicity is critically important, as it acknowledges not only that the construction of gendered identities is racialized, but also that feelings of feminine typicality and masculine typicality may have different implications for attachment to a field dominated by White men.

## 4. Materials and Methods

### 4.1. Participants and Procedure

The quantitative survey data utilized for this study come from a larger IRB-approved and NSF-funded research project on young women’s engineering experiences. Survey participants are undergraduate women in the United States who are collegiate members of the Society of Women Engineers (SWE), which is an engineering professional organization that supports women in engineering and technology fields. As SWE collegiate members, the young women can select to sign up to be members of the local SWE group at their college or university, as well as participate at the national level, at any point during their undergraduate years. Through their collaboration with SWE on the broader research project, the authors had access to SWE’s listserv and contacted SWE collegiate members to participate in the study via email. 

We collected cross-sectional survey data in Spring 2019, 2020, and 2021. Our analytic sample includes 804 SWE collegiate members from across the U.S. who self-identified as women/female and had declared a major in any engineering discipline. We note that our survey question about gender identity included categories for women/female, man/male, or other gender (where respondents could write in their chosen identity); only 7 respondents identified as male/men, and 11 wrote in their gender identity, which included non-binary and gender fluid. Unfortunately, given the very small number of respondents in these groups, they were dropped from the analysis due to the inability to estimate models with our categorical dependent variables (i.e., respondents were absent from several categories; therefore, odds could not be estimated). Regarding race/ethnicity, respondents were asked to self-report, which included a selection of any or multiple racial/ethnic identities, such as American Indian or Alaska Native (AI/AN), Asian, Black, Latinx, Native Hawaiian or Pacific Islander (NH/PI), and/or White. A composite race/ethnicity variable was constructed for use in our subsequent analysis to distinguish between Asian, Black, Latinx, multiracial, and White women, and multiracial women included those who had self-identified with two or more racial/ethnic identities; we note that almost all students who identified as AI/AN or NH/PI also identified with one or more additional categories and are therefore included in the multi-racial category; the few cases remaining were not included in the analysis due to problems with estimation in multivariate models. In all, the final analytic sample includes 11.8% Asian, 3.6% Black, 9.5% Latinx, 6.6% multiracial, and 68.5% White women; these percentages are similar to the racial/ethnic representation among women earning bachelor’s degrees in engineering nationwide [42].

In our sample, participants report majoring in an array of different engineering subdomains, including aerospace, chemical, civil, electrical, and mechanical engineering. The young women engineers are generally very high-achieving, as indicated by their self-reported GPA, and the large majority report having college-educated mothers. We include variables to measure all of the aforementioned sample characteristics in our multivariate models and, therefore, discuss them in more detail in the section for additional variables (and also see Table 1).

### 4.2. Authors’ Positionality

The first author of this study is an assistant professor of education and identifies as a Latinx–Asian cisgender woman who graduated with a B.S. degree in engineering and a doctoral degree in education. She considers herself a primarily quantitative researcher who explores issues of equity in STEM education, with a specific focus on historically minoritized learners, such as young women and Students of Color. Her lived experiences as a Woman of Color in engineering and a PK-16 STEM educator helped inform the quantitative analytic process. The second author is a professor of sociology and education who identifies as a White cisgender woman who has conducted research for many years on gender and racial inequality in STEM education. In engaging in this research work, they are aware that while their individual experiences provide insight for interpreting the analytic results, these perspectives may be limited and can lead to oversights. Therefore, the authors have carefully reflected and considered alternative interpretations of the results through discussions with equity-focused STEM education colleagues and scholars. 

### 4.3. Measures

#### 4.3.1. Measures of Gender Typicality

Our first research question focuses on how young women engineers define their gender typicality, defined as described below.

Feminine typicality. Our measure captures how young women perceive themselves as typical in relation to other young women and it was adapted from a previously validated scale by Martin et al. [5] to better reflect the age of the participants, such that any mention of ‘girls’ was replaced with ‘women my age.’ This scale variable was constructed by averaging across four items, and response categories for these items ranged from 1 (strongly disagree) to 5 (strongly agree). Items comprising the feminine typicality scale variable were: “I feel similar to women my age”, “I act like women my age”, “I like to do the same things as women my age”, and “I like to spend time with women my age”. 

Masculine typicality. Likewise, this scale captures the extent to which young women perceive themselves as typical of men, including in their behaviors and actions [5]. We replaced instances of ‘boys’ in the original items with ‘men my age.’ The scale variable was constructed by averaging across four items, with responses ranging from 1 (strongly disagree) to 5 (strongly agree). Parallel items were used for masculine typicality, including: “I feel similar to men my age”, “I act like men my age”, “I like to do the same things as men my age”, and “I like to spend time with men my age”. 

A confirmatory factor analysis (CFA) was conducted to confirm the factor structure for feminine typicality and masculine typicality. Beginning with feminine typicality, indicators examined showed an adequate model fit ($\chi^{2}$(2) = 13.32, *p* < 0.01, CFI = 0.990, RMSEA = 0.075 (90% CI [0.040, 0.115]), SRMR = 0.020) [43,44]. All standardized factor loadings (coefficients) for feminine typicality ranged between 0.56 and 0.77 and were significant (*p* < 0.001). The indicators for masculine typicality also suggested an adequate model fit ($\chi^{2}$(2) = 23.39, *p* < 0.001, CFI = 0.969, RMSEA = 0.103 (90% CI [0.068, 0.142]), SRMR = 0.031), and all standardized factor loadings were significant (*p* < 0.001) and ranged between 0.47 and 0.70. Moreover, Cronbach’s alpha values for feminine typicality and masculine typicality were 0.79 and 0.70, respectively, suggesting good internal reliability for both scale variables.

#### 4.3.2. Measures of Engineering Attachment

Our second research question focuses on how gender typicality predicts young women engineers’ attachment to the field, which we measure with two different variables. ‘Engineering identity’ captures the extent to which young women perceive a more personal and proximal connection to engineering and, more specifically, as participants in the field of engineering. And, as described below, ‘commitment to engineering major’ captures how certain young women are that they will stay in the field. As the correlation between these two outcomes is small, *r*(802) = 0.34, *p* < 0.001, they represent distinct measures of young women’s attachment to engineering.

Engineering identity. Participants were asked to report their level of agreement with the statement, “I see myself as an engineering person”. This item is commonly used by STEM education scholars [45,46] and is an adaptation of mathematics and science identity measures from national longitudinal studies (see High School Longitudinal Study of 2009 [47]). The original ordinal variable ranged from 1 (strongly disagree) to 5 (strongly agree). Due to small cell sizes, we combined the lowest two categories, resulting in a four-category ordinal variable. However, we note that analysis using the original five-category variable as the dependent variable (utilizing ordered logit models), as well as analysis using a dichotomized variable (utilizing logistic regression) yielded highly comparable results to those reported for our models below in the results section.

Commitment to engineering major. Participants were asked to indicate how often they thought about changing their major on a scale from 1 (never) to 7 (almost always). This item has been utilized in prior research to examine undergraduate’s intentions to remain in STEM fields [48]. We reverse-coded the variable such that high values capture commitment to the major (e.g., never considering changing their major), and lower values capture frequent thoughts of leaving. Due to small cell sizes, we collapsed the categories for the most frequent consideration of leaving, resulting in a five-category ordinal variable. Ordered logit models with the original seven-category variable as the dependent measure, as well as logistic models with a dichotomous measure, yielded highly comparable results to those described below.

#### 4.3.3. Additional Variables

Race and SES. As described earlier, our survey includes measures of self-reported race/ethnicity, and these racial/ethnic groups include Asian, Black, Latinx, multiracial, and White. These categorical indicators are included in our multivariate models (described below). We also include the mother’s highest level of education as a proxy for socio-economic status (*SES*) based on prior research on its reliability and validity [49,50,51]. Specifically, the women in our sample reported their mother’s highest level of education by selecting from the following categories: less than a high school diploma, high school diploma, associate’s degree, bachelor’s degree, master’s degree; or PhD, MD, or law degree. As most participants reported having college-educated mothers, we subsequently created a dichotomous variable to differentiate between those whose mothers’ highest level of education was at least a bachelor’s degree (68.5%) and those whose mothers’ highest level of education was less than a bachelor’s degree (31.5%). 

Engineering major composition. Because women’s representation in engineering varies across different subfields, which may have implications for their feelings of fit and attachment, we include measures of gender composition as control variables in our multivariate analysis. Utilizing national data on women’s representation from [42], we distinguish between engineering subfields that have a low proportion of women (less than 30% women), and a higher proportion of women (greater than or equal to 30% women). We included a third category for those whose gender composition could not be determined as the choice of subfield selected was ‘other’ engineering (other engineering major subfields). Engineering majors with less than 30% women include: aerospace engineering, computer engineering, electrical engineering, mechanical engineering, and petroleum engineering. Engineering disciplines with at least 30% women include: architectural engineering, biomedical engineering, chemical engineering, civil engineering, and environmental engineering. 

Other control variables: year in college, GPA, and survey cohort. Finally, our multivariate models included several other control variables to ensure the robustness of our models predicting attachment to engineering. ‘Upper-class student’ differentiates between young women who were at least in their third year of college and those who were in their first or second year of college. Additionally, ‘high GPA’ distinguishes young women with a self-reported GPA of at least 3.50 and those without. ‘Cohort’ is a categorical variable that distinguishes between young women who completed the survey in Spring 2019, 2020, or 2021. See Table 1 for descriptive statistics of the variables discussed above.

### 4.4. Analytic Method

To address our first research question, how women college engineers define their gender typicality along feminine and masculine dimensions, and how this varies by race/ethnicity, we first calculated overall means of both typicality measures and then tested for racial/ethnic differences with ANOVA. We also calculated correlations between masculine and feminine typicality measures overall and separately by racial/ethnic group. This provides important context before moving to our multivariate models.

Specifically, our second research question asks how perceptions of feminine typicality and masculine typicality predict attachment to engineering, and how this varies by race/ethnicity. As our dependent measures capturing engineering attachment (engineering identity and commitment to major) are both ordinal, we performed ordered logit models, after first conducting Brant’s tests to confirm that assumptions of proportional odds were not violated [52]. Analyses for each dependent variable begin with baseline models (Model 1) including only measures of gender typicality, and then add background and control variables (Model 2). Finally, to address how relationships may vary by race/ethnicity, we add interaction terms between women’s racial/ethnic identity and their perceptions of both feminine typicality and masculine typicality (Model 3). Given their position of racial privilege, White women serve as the reference category when estimating both the main effects of race/ethnicity and interactions with measures of gender typicality; however, we note that no additional statistically significant interactions were found when the reference category was changed.

## 5. Results

### 5.1. Examining Feminine and Masculine Typicality

**Figure 1:**
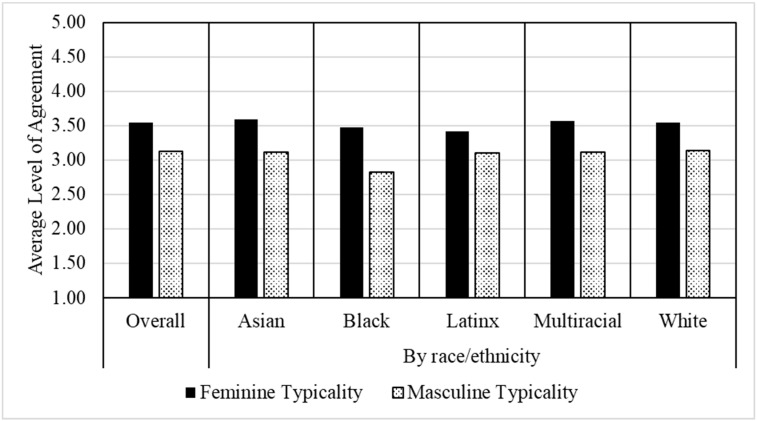
Average scores on scales measuring feminine typicality and masculine typicality.

Figure 1 shows the means for the measures of gender typicality. We include means for the overall sample as well as by women’s race/ethnicity, where higher values on feminine and masculine typicality indicate higher levels of agreement of fit with young women and young men, respectively. Among our sample of women college engineers, the average feminine typicality was 3.54 (*SD* = 0.74). That is, they consider themselves to be somewhat typical of women. Participants also expressed a moderate degree of feeling typical of men, as the mean on this dimension was 3.12 (*SD* = 0.68). Yet respondents reported significantly lower levels of masculine typicality compared to feminine typicality, *t*(803) = 13.89, *p* < 0.001), and this difference was moderate in size (*Cohen’s d* = 0.59). 

We also observed a small but significant positive correlation between feminine typicality and masculine typicality, *r*(802) = 0.28, *p* < 0.001. Interestingly, this departs from previous research on dual gender typicality, which has found small but negative correlations between the two scales [5,30]. Yet these studies used ‘general’ samples (i.e., not specific to a certain group); given that our sample is purposively limited to women engineers, this perhaps suggests that they are more likely than the general population of men and women to consider femininity and masculinity as dimensions that can co-exist and are not necessarily contradictory.

Regarding potential differences by race/ethnicity, we conducted one-way ANOVA tests, which revealed no significant differences across groups on means for either feminine typicality, *F*(4, 799) = 0.72, *p* = 0.58, or masculine typicality, *F*(4, 799) = 1.57, *p* = 0.18. Yet, as shown in Table 2, we did observe some fluctuations between groups regarding the correlations between the two scales, such that while the correlation for White women was close to the sample average, *r*(549) = 0.33, *p* < 0.001, it was stronger for Latinx, *r*(74) = 0.53, *p* < 0.001, and Black women, *r*(27) = 0.48, *p* < 0.01, and non-significant and close to zero for multiracial, *r*(51) = 0.02, *p* > 0.05, and Asian women, *r*(93) = −0.10, *p* > 0.05. This suggests that how women view masculinity and femininity, and the extent to which they see them as complementary or more independent dimensions of gender identity, does indeed vary by racial/ethnic identity.

### 5.2. Predicting Engineering Identity

Results from ordered logit models predicting engineering identity are found in Table 3, displayed as odds ratios, such that values greater than one indicate a positive association and values less than one indicate a negative association. As shown in Model 1, there is no statistically significant effect of feminine typicality in predicting engineering identity. Yet masculine typicality is positively and significantly associated with engineering identity. Specifically, a one-unit increase in masculine typicality increases young women’s odds of having a higher level of engineering identity by a factor of 1.424 (*p* < 0.001, 95% CI [1.16, 1.74]). In Model 2, the effect of masculine typicality remains significant and robust with the inclusion of background and control variables (OR = 1.392, *p* < 0.01, 95% CI [1.13, 1.72]).

To further illustrate the observed associations, we calculated predicted probabilities (using the ‘margins’ post-estimation command in Stata) of having the lowest level of engineering identity, and then alternatively, the highest levels of engineering identity for respondents who reported different levels of masculine typicality, holding all other variables to the mean. For example, while overall, few respondents are in the lowest level of engineering identity (as seen in Table 1), the probability of being in this category decreases as masculine typicality increases, going from 0.063 for those at the minimum value of masculine typicality to a probability of only 0.018 for those at the maximum value. The opposite pattern is observed at the highest level of engineering identity (which is overall more common among the sample), such that the probability of having the strongest level of identity increases as masculine typicality increases, going from 0.244 for those who report the lowest masculine typicality to a probability of 0.547 for those who report the highest masculine typicality.

Returning to other results as shown in Model 2, we did not observe any significant differences in engineering identity by women’s race/ethnicity. However, in our sample, women of higher SES have significantly lower engineering identities than women of lower SES. Holding all else constant, the odds of having a higher level of engineering identity are lower for those women from a higher SES by a factor of 0.649 (*p* < 0.01, 95% CI [0.48, 0.87]). Additionally, women in engineering majors with very low proportions of women (less than 30%) had significantly stronger engineering identities than those in engineering majors with higher proportions of women (OR = 0.669, *p* < 0.01, 95% CI [0.50, 0.90]), and those in other engineering majors (OR = 0.547, *p* < 0.01, 95% CI [0.37, 0.80]), as evidenced by the odds ratios in the latter two categories being less than one in relation to the reference category. 

Finally, as shown in Model 3, there are no significant interactions between either feminine typicality or masculine typicality and women’s race/ethnicity. Put differently, feelings of feminine typicality are independent of engineering identity (e.g., not significantly associated) for women across different racial/ethnic groups. Additionally, stronger perceptions of fit and similarity with men (e.g., masculine typicality) are similarly predictive of stronger engineering identity for young women from different racial/ethnic groups (OR = 1.453, *p* < 0.01, 95% CI [1.12, 1.88]).

### 5.3. Predicting Commitment to Engineering Major

Now, we turn to the results of ordered logit models displaying the association between gender typicality and young women’s commitment to staying in engineering, as shown in Table 4. Beginning with Model 1, feminine typicality is significantly and negatively associated with engineering major commitment, such that higher levels predict significantly lower levels of commitment to engineering (OR = 0.784, *p* < 0.01, 95% CI [0.66, 0.94]). In contrast, masculine typicality is positively and significantly associated with commitment to their engineering major (OR = 1.285, *p* < 0.05, 95% CI [1.06, 1.56]). In Model 2, both of these effects remain significant and robust with the inclusion of all background and control variables.

To better illustrate the opposite directions of these associations, we again calculated predicted probabilities of being in the lowest and highest levels of the dependent variable, in this case, commitment to engineering major, based on varying levels of gender typicality. Beginning with feminine typicality, the probability of having the lowest level of engineering commitment increases as feminine typicality increases, going from a probability of 0.079 for those at the minimum level of femininity to a probability of 0.188 for those at the maximum. In contrast, as masculine typicality increases from the minimum to the maximum values, the probability of having the lowest level of engineering commitment decreases from 0.213 to 0.093. Turning to the probabilities of being in the highest category of engineering commitment, the probability decreases as feminine typicality increases from the minimum to the maximum values, going from a probability of 0.368 to a probability of 0.179. Yet as masculine typicality increases across the same range, the probability of having the highest level of engineering commitment increases from 0.157 to 0.331.

Returning to associations between engineering commitment and other variables as shown in Table 4, we see that relative to White women, Asian women (OR = 0.633, *p* < 0.05, 95% CI [0.42, 0.95]) and multiracial women (OR = 0.591, *p* < 0.05, 95% CI [0.36, 0.98]) report significantly lower levels of commitment to staying in the major. Women with high GPA (>3.5) have higher levels of commitment relative to women with lower GPA, but this result is only significant at the alpha = 0.10 level (OR = 1.290, *p* = 0.057, 95% CI [0.99, 1.68]).

Finally, analyses in Model 3 examine possible differentiation by women’s race/ethnicity in the relationship between gender typicality and engineering commitment. There is no significant interaction between race/ethnicity and masculine typicality. That is, the positive association between masculine typicality and engineering commitment is consistent across women from different racial/ethnic groups. Yet interestingly, we observe a positive and statistically significant interaction for Latinx women with feminine typicality. The odds ratio (OR = 2.006, *p* < 0.05, 95% CI [1.01, 3.96]) indicates that in contrast to the reference group of White women, for Latinx women, higher levels of feminine typicality are associated with stronger engineering commitment.

To better illustrate these associations, we report predicted probabilities of being in the lowest and highest levels of engineering commitment separately for White women (the reference group) and Latinx women, as feminine typicality increases. Beginning with the former, we note that the probabilities for White women closely track those of the aggregate sample captured by the main effect of feminine typicality as described above (which is consistent with the fact that they comprise the majority of the sample, and that there are no other significant racial/ethnic interactions with feminine typicality). Specifically, for White women, the probability of having the lowest level of engineering commitment is higher (probability = 0.194) when feminine typicality is very high, compared to when it is very low (probability = 0.054). And for White women, the probability of having the highest level of engineering commitment decreases as feminine typicality increases, going from 0.465 to 0.172. Yet the pattern is reversed for Latinx women, such that the probability of having the lowest level of engineering commitment is highest when feminine typicality is very low (probability = 0.283) than when it is very high (probability = 0.093). Moreover, the probability of having the highest engineering commitment increases for Latinx women along with increasing levels of feminine typicality, from a low probability of 0.112 when femininity is very low, to a probability of 0.328 when feminine typicality is very high. In sum, we find a unique pattern for Latinx women, such that feminine typicality predicts significantly stronger engineering commitment, while for other women, it predicts significantly weaker commitment.

## 6. Discussion

Women continue to be under-represented in many high-status and high-income STEM occupations, including engineering [1]. The much lower likelihood of women entering engineering majors in college contributes greatly to their under-representation in such occupations, and as such a large extant body of research focuses on the factors that deter them from making this choice (e.g., Cheryan and Markus [2]). Yet given that so few women enter these majors in the first place, any attrition is a major concern. As such, this study chose to focus on a unique sample of young women who have already chosen to enter engineering, to explore their attachment to their field, and the factors that might support or undermine it.

Specifically, guided by recent theoretical and empirical research that recognizes gender identity as multi-dimensional [5,16] and prior qualitative work on women in STEM [37,39], we addressed two research questions. First, how do women college engineers define their gender typicality along feminine and masculine dimensions, and how does this vary by race/ethnicity? Second, how do their perceptions of feminine typicality and masculine typicality predict their attachment to engineering, including their engineering identity and intentions to stay in the field; and how does this vary by race/ethnicity? 

Notably, our study departs from prior research that utilizes a single measure of gender typicality (i.e., own-gender typicality), measures of identity compatibility, or views gender typicality as a continuum (and effectively conceptualizes femininity and masculinity as diametrically opposed) [7,27,33,35]. Instead, our study contributes to the emerging dual gender typicality perspective by providing quantitative evidence regarding identification with feminine typicality and masculine typicality among women engineers and related implications for their attachment to their chosen field. Importantly, our work is informed by intersectional scholars [8,39], and calls attention to the need to consider how gender identities, including perceptions of feminine and masculine typicality, are likely constructed differently across racial/ethnic communities and, as such, may have different implications for educational and occupational pathways.

### 6.1. Patterns of Gender Typicality

In regard to the results of our first research question, our study supports the claim of some recent research that feminine typicality and masculine typicality are distinct dimensions of gender identity that should be considered simultaneously [5,30], as the women college engineers in our study express some compatibility with both feminine typicality and masculine typicality (both means are around the midpoint of their respective scales). Further, our descriptive results indicate a positive correlation (*r* = 0.28) between these measures, which differs from the slight negative association observed with more general samples of children and young adults [5,30]. This suggests that perhaps among young women who have entered men-dominated fields, feminine typicality and masculine typicality are not necessarily seen as incompatible or opposing dimensions of gender identity, but rather can be complementary. Moreover, consistent with some previous qualitative research, they may be flexibly navigating between femininity and masculinity [37,38]. This potential positive pairing of masculinity and femininity appears to be even more pronounced for Black and Latinx women engineers than their White peers, as the correlations were relatively stronger for the former groups (*r* = 0.48 and 0.53, respectively) than the latter (*r* = 0.33); yet, for Asian and multiracial women in our study, such positive associations are absent. Such patterns perhaps echo previous research findings that perceptions of masculine and feminine traits may be racialized [53]. From our perspective, these descriptive results offer a clear impetus for future research to continue to explore dimensions of gender identity, including feminine typicality and masculine typicality among young women in a variety of contexts, with focused attention on variation by racialized identities.

### 6.2. Considering the Privileged Role of Masculine Typicality in Engineering

Our second research question explored whether and how masculine and feminine typicality predicted young women engineers’ attachment to engineering, and whether and how such associations varied by race/ethnicity. We indeed found evidence that both typicality measures mattered, albeit in different ways, and there was some important variation across racial/ethnic groups. Beginning with results for masculine typicality, overall, we found that it positively and significantly predicted attachment to engineering. Specifically, expressing higher ratings of masculine typicality is associated with a stronger engineering identity, as well as a stronger commitment to staying in an engineering major; these findings are consistent across women from different racial/ethnic groups. Taken together, these results suggest that connection to a heteronormative masculine field, such as engineering, is generally stronger among women who perceive themselves as having strong similarity to men. This finding is consistent with prior qualitative work that has found that women who identify with masculine traits and behaviors tend to feel more connected to STEM fields [38,40,54].

### 6.3. Considering the Role of Feminine Typicality in Engineering

The results from analyses examining the relationship between feminine typicality and women’s connection to engineering reveal a somewhat more complicated story, as we found that it did not significantly predict engineering identity, but it did significantly predict commitment to major; however, this later finding also diverged by race/ethnicity. Beginning with the null finding for engineering identity, this suggests that women college engineers can form a strong engineering identity independent of how typical they feel of their own gender. This result stands somewhat in contrast to prior research which presumes that women in STEM perceive incompatibility between their gender identity and immediate identification as a STEM person [32,36]. Therefore, our study challenges the notion that femininity is consistently incompatible or interferes with engineering identity.

Nonetheless, feminine typicality negatively predicted commitment or certitude of staying in their engineering major for women in our study (with the exception of Latinx women who we will turn to discuss next). So how do we make sense of the fact that feminine typicality is independent of engineering identity, yet negatively predicts commitment to major for women from most racial/ethnic groups? Perhaps this is due to engineering identity capturing a belief about personally fitting with engineering as a vocation, while commitment to major may be capturing not just fit with engineering, but perceived fit in relation to other fields (e.g., feeling like there are other areas where one could be successful). Put differently, women with higher feminine typicality may feel that their expressions of femininity are not recognized by others as compatible with engineering or are devalued by others in engineering spaces [37,55] and may view non-engineering fields as more compatible or welcoming to feminine women [56,57]. We argue that this finding warrants further examination of the complexity in how women engineers see the potential benefits and disadvantages of feminine typicality in different contexts.

Relatedly, we find interesting racial/ethnic variations in patterns for how feminine typicality predicts commitment to major, as for Latinx women, it is in fact a positive predictor. This result offers evidence in support of the notion that femininity (and masculinity) are constructed and enacted differently across racial/ethnic communities. In this case, literature on the strong familial focus of Latinx communities may be relevant, as well as research on the cultural expectations of caretaking roles as expressed by young college Latinas [58,59]. For example, research by Ovink [60] suggests that Latinas view their college pathways as familial rather than individual ‘investments.’ From this lens, for Latinx women, a feminine identity may be partly characterized as persevering for the ultimate benefit of their family, and thus remaining committed to completing an engineering degree once begun (particularly given the likely lucrative nature of such a major). Our results are necessarily speculative; however, they speak to the need for more research on how gender identities are constructed and performed across different communities.

### 6.4. Limitations and Future Directions

Although this study provides new insights into the relationship between engineering women’s gender typicality and their perceived connection to engineering, we recognize several limitations. First, the data for this study are cross-sectional, and we do not claim to make any causal inferences. As such, future longitudinal studies should examine how women’s identification with feminine typicality and masculine typicality may change throughout their undergraduate studies in engineering as well as how these are recognized by others as legitimate or compatible with a future in engineering. Moreover, the young women in this study are self-selected participants of the Society of Women Engineers (SWE), an engineering professional organization. Therefore, the results may not be generalizable to all college women studying engineering. 

Additionally, while our study recognizes gender as multi-dimensional, it is limited by its absence of non-binary and transgender individuals. Clearly, this should be a priority for future research, as the few recent studies that examine their experiences in STEM fields point to complex ways in which masculinity is privileged. For example, while discrimination and exclusion of non-traditional gender identities are certainly pervasive, Alfrey and Twine [54] find that women in tech companies who identify as gender fluid are viewed as more competent than other women by male colleagues, and Kersey and Voight [61] report a similar pattern for transgender men in STEM fields in academia. Including cisgender men in examinations of how different dimensions of gender identity (including perceived masculine and feminine typicality) are more or less advantageous in STEM settings would also provide more insights. Further, our study did not examine the potential role of sexual orientation, which scholars point to as another dimension of gender identity with implications for power and inequality in STEM fields (see work by Levya et al. [62] and Cech [63]). 

And finally, while our study includes a racially/ethnically diverse sample of engineering women from across the U.S., and highlights the importance of considering racial identities, it cannot speak directly to how women from different groups define and perform femininity and masculinity, nor how it is recognized or perceived by those with privilege in hegemonically masculine STEM spaces [54]. We hope that future research might focus on such issues.

## 7. Conclusions

This study underscores the importance of utilizing a multi-dimensional perspective of gender identity to examine the experiences of women, particularly in highly gendered learning environments such as engineering, as well as considering racial/ethnic variation. To our knowledge, this is the first quantitative study to provide empirical evidence of the presence and implications of dual measures of gender typicality, namely feminine typicality and masculine typicality, among a U.S. sample of racially diverse women in men-dominated STEM fields. While our findings support other related research on the privilege of masculinity in such spaces, albeit measured quite differently, they also complicate a simple story that presumes that femininity may be consistently devalued. Relatedly, our study supports the calls of intersectional scholars to acknowledge and explore meaningful differences in the construction of different aspects of gender identities across different communities. In closing, we hope that future efforts to improve gender equity in engineering will focus on changing the real and perceived culture of the field as one where all gender identities are recognized and welcome.

## Figures and Tables

**Table 1:** Sample characteristics.

	Percentage
Attachment to Engineering	
Engineering identity	
(1) Lowest level of engineering identity	3.48%
(2)	10.95%
(3)	45.77%
(4) Highest level of engineering identity	39.80%
Commitment to engineering major (Reverse-coded)	
(1) Lowest level of engineering commitment	14.43%
(2)	11.32%
(3)	16.04%
(4)	33.83%
(5) Highest level of engineering commitment	24.38%
Background Variables	
Race/Ethnicity	
Asian	11.82%
Black	3.61%
Latinx	9.45%
Multiracial	6.59%
White	68.53%
Mother’s highest level of education (SES proxy)	
Less than a bachelor’s degree	31.47%
At least a bachelor’s degree	68.53%
Engineering major composition	
Low proportion of women (<30% women)	44.40%
Higher proportion of women (≥30%)	39.18%
Other engineering major subfields	16.42%
Upper-class student (3+ years in college)	63.43%
High GPA (3.50+)	59.83%
Cohort	
2019	34.58%
2020	33.83%
2021	31.59%
N	804

**Table 2:** Correlations between feminine and masculine gender typicality measures.

	Overall Sample	By Race/Ethnicity				
		Asian	Black	Latinx	Multiracial	White
*Correlation Coefficient*	0.28 ***	−0.10	0.48 **	0.53 ***	0.02	0.33 ***

*** *p* < 0.001, ** *p* < 0.01.

**Table 3:** Results of ordered logit models predicting women’s engineering identity.

	Model 1	Model 2	Model 3
Key Independent Variables			
Feminine typicality	0.884	0.942	0.883
	(0.084)	(0.091)	(0.105)
Masculine typicality	1.424 ***	1.392 **	1.453 **
	(0.147)	(0.149)	(0.190)
Interactions			
Race/Ethnicity × Feminine typicality (Ref: White × Feminine typicality)			
Asian × Feminine typicality			0.997
			(0.273)
Black × Feminine typicality			1.296
			(0.865)
Latinx × Feminine typicality			1.584
			(0.567)
Multiracial × Feminine typicality			1.327
			(0.546)
Race/Ethnicity × Masculine typicality (Ref: White × Masculine typicality)			
Asian × Masculine typicality			0.880
			(0.298)
Black × Masculine typicality			0.666
			(0.362)
Latinx × Masculine typicality			0.893
			(0.335)
Multiracial × Masculine typicality			0.893
			(0.391)
Background Variables			
Race/Ethnicity (Ref: White)			
Asian		0.859	1.293
		(0.183)	(1.896)
Black		0.739	0.953
		(0.262)	(2.101)
Latinx		0.732	0.215
		(0.175)	(0.273)
Multiracial		0.778	0.404
		(0.216)	(0.793)
SES (Ref: Low SES)			
High SES		0.649 **	0.647 **
		(0.097)	(0.098)
Engineering major composition (Ref: Low proportion of women, < 30%)			
Higher proportion of women (≥ 30%)		0.669 **	0.667 **
		(0.100)	(0.100)
Other engineering major subfields		0.547 **	0.545 **
		(0.107)	(0.107)
3+ years in college (Ref: less than 3 years)		1.103	1.103
		(0.156)	(0.158)
High GPA (Ref: GPA less than 3.5)		1.087	1.096
		(0.155)	(0.157)
Cohort (Ref: 2019 cohort)			
2020 cohort		1.024	1.024
		(0.173)	(0.174)
2021 cohort		1.049	1.056
		(0.180)	(0.182)
Cutoff 1	0.069 ***	0.046 ***	0.042 ***
	(0.030)	(0.023)	(0.023)
Cutoff 2	0.322 **	0.220 **	0.200 **
	(0.130)	(0.103)	(0.106)
Cutoff 3	2.961 **	2.137	1.951
	(1.195)	(0.998)	(1.034)

Notes: N = 804; odds ratios are from ordered logit models; robust standard errors are in parentheses; *** *p* < 0.001, ** *p* < 0.01.

**Table 4:** Results of ordered logit models predicting women’s commitment to engineering major.

	Model 1	Model 2	Model 3
Key Independent Variables			
Feminine typicality	0.784 **	0.783 **	0.699 **
	(0.071)	(0.071)	(0.079)
Masculine typicality	1.285 *	1.277 *	1.382 **
	(0.125)	(0.128)	(0.173)
Interactions			
Race/Ethnicity × Feminine typicality (Ref: White × Feminine typicality)			
Asian × Feminine typicality			1.300
			(0.344)
Black × Feminine typicality			1.045
			(0.716)
Latinx × Feminine typicality			2.006 *
			(0.697)
Multiracial × Feminine typicality			1.031
			(0.392)
Race/Ethnicity × Masculine typicality (Ref: White × Masculine typicality)			
Asian × Masculine typicality			0.784
			(0.246)
Black × Masculine typicality			1.192
			(0.668)
Latinx × Masculine typicality			0.644
			(0.233)
Multiracial × Masculine typicality			0.881
			(0.355)
Background Variables			
Race/Ethnicity (Ref: White)			
Asian		0.633 *	0.534
		(0.129)	(0.764)
Black		0.748	0.394
		(0.258)	(0.788)
Latinx		0.812	0.287
		(0.185)	(0.341)
Multiracial		0.591 *	0.783
		(0.153)	(1.525)
SES (Ref: Low SES)			
High SES		0.800	0.805
		(0.114)	(0.116)
Engineering major composition (Ref: Low proportion of women, <30%)			
Higher proportion of women (≥30%)		1.176	1.177
		(0.164)	(0.164)
Other engineering major subfields		1.087	1.079
		(0.203)	(0.203)
3+ years in college (Ref: less than 3 years)		1.186	1.193
		(0.159)	(0.161)
High GPA (Ref: GPA less than 3.5)		1.290 ~	1.274 ~
		(0.172)	(0.172)
Cohort (Ref: 2019 cohort)			
2020 cohort		0.956	0.971
		(0.150)	(0.154)
2021 cohort		1.072	1.094
		(0.176)	(0.180)
Cutoff 1	0.154 ***	0.157 ***	0.135 ***
	(0.059)	(0.068)	(0.067)
Cutoff 2	0.318 **	0.328 **	0.283 *
	(0.120)	(0.142)	(0.139)
Cutoff 3	0.665	0.693	0.600
	(0.251)	(0.299)	(0.293)
Cutoff 4	2.912 **	3.102 **	2.705 *
	(1.102)	(1.342)	(1.323)

Notes: N = 804; odds ratios are from ordered logit models; robust standard errors are in parentheses; *** *p* < 0.001, ** *p* < 0.01, * *p* < 0.05, ~ *p* < 0.10.

## Data Availability

The data presented in this study are available on request from the corresponding author.
